# Data on manmade sinkholes due to leakage in underground pipelines in different subsurface soil profiles

**DOI:** 10.1016/j.dib.2021.106740

**Published:** 2021-01-09

**Authors:** Haibat Ali, Jae-ho Choi

**Affiliations:** Department of Civil Engineering, Dong-A University, 550 Bungil 37, Nakdong-Daero, Saha-Gu, Busan 49315, South Korea

**Keywords:** Sinkhole, Risk prediction, Sewer pipeline, Pipeline leakage, Soil profile

## Abstract

This paper provides simulated datasets for different versions of small-scale physical sinkhole models that are essential to understand the sinkhole formation rate. These physical models were used in experiments to monitor ground settlement or collapse due to leakage from an underground pipeline. The factors under consideration were the subsurface soil profile, pattern of water flow, and leakage position in the pipeline. The experimental results and statistical analysis showed that the subsurface soil strata conditions dominated the sinkhole occurrence mechanism, although other factors also contributed to the settlement. The results also showed that the subsurface soil comprising strata sandy clay, limestone, and bedrock (SC-LS-BR) dominates the sinkhole mechanism. The data are organized and formated in a useful structure. Specifically, the dataset is presented in terms of tables to illustrate the settlements in different soil profiles under various conditions. This analysis was then used to predict the sinkhole risk level under different conditions. The formulated dataset and the results can be considered in developing a sinkhole risk index (SRI) and identifying sinkhole risk areas.

## Specifications Table

**Table:** 

Subject	Civil Engineering
Specific subject area	Urban Sinkhole Risk Reduction, Sustainable Urban Development.
Type of data	Excel spreadsheets, table.
How data were acquired	Systematic experiments were conducted to monitor the settlement and collapse of different subsurface soil profiles under various leaking conditions of underground water pipelines.
Data format	Raw and analyzed.
Parameters for data collection	The type of flow inside the pipeline, type of soil strata, and time interval are considered as parameters to measure the soil settlement or collapse to create a sinkhole.
Description of data collection	Data was collected manually by reading the settlement changes from the measuring tape attached to each experimental setup. The measuring tape was attached vertically in each case to the experimental box. The data was collected every 60 s.
Data source location	Institute: Dong-A University City: Busan Country: South Korea
Data accessibility	RAW data from Mendeley Data, “Data on manmade sinkholes due to leakage in underground pipelines in different subsurface soil profiles,” Mendeley Data, V1, DOI: 10.17632/7mgtzphnd2.1
Related research article	The associated data is from H. Ali and J.H. Choi, 2019, Risk Prediction of Sinkhole Occurrence for Different Subsurface Soil Profiles Due to Leakage from Underground Sewer and Water Pipelines. Sustainability (2019), DOI: 10.3390/su12010310

## Value of the Data

• A dataset of manmade sinkholes (see [2]) generated from leaking pipelines in different subsurface soil profiles can be useful for predicting the sinkhole risk in urban areas.
• This dataset can help public and private maintenance authorities, such as urban water management authorities and geological departments, to take action as soon as possible to prevent accidents due to leaking underground sewers and/or water pipelines.
• Researchers in the area of urban disaster risk prediction and urban infrastructure development can use the data to develop a Sinkhole Risk Index (SRI).

## 1 Data Description

The presented data (see [2]) were obtained from a systematic experimental investigation of manmade sinkholes due to leakage in underground water pipelines in different subsurface soil profiles. Two different types of water flow, continuous and non-continuous flows, were considered. The subsurface soil profiles under consideration were:

1. Sandy clay (SC)
2. Sandy clay and Bedrock (SC-BR)
3. Sandy clay, Limestone, and Bedrock (SC-LS-BR)
4. Sandy clay, Cavity, and Bedrock (SC-C-BR)

Table 1 outlines the settlement data for four different soil profiles when the flow inside the pipeline was continuous.

**Table 1:** Settlement values for continuous water flow through four different soil profiles.

Time (s)	Settlement (mm)			
	Case I (a)- SC	Case I (b)- SC-BR	Case I (d)- SC-LS-BR	Case I (c)- SC-C-BR
60	0	0	0	0
120	0	0	0	0
180	0	0	0	0
240	0	0	0	0
300	0	0	0	0
360	0	1	1	1
420	1	3	3	3
480	2	4	4	4
540	3	5	7	7
600	4	6	9	10
660	4	7	14	15
720	5	9	17	17
780	6	11	21	22
840	7	13	25	25
900	8	15	29	110
960	9	18	32	111
1020	10	21	34	113
1080	11	25	36	115
1140	12	29	139	116
1200	14	33	140	118
1260	17	37	142	120
1320	18	39	144	122
1380	19	42	148	123
1440	20	45	153	124
1500	21	47	154	125
1560	22	49	155	127
1620	23	51	157	130
1680	24	53	158	133
1740	25	55	159	134
1800	26	57	160	135

Table 2 shows the settlement data for four different soil profiles when the flow inside the pipeline was non-continuous (30 s cyclic time interval).

**Table 2:** Settlement values for non-continuous water flow with 30 s cyclic time interval through four different soil profiles.

Time (s)	Settlement (mm)			
	Case II (a)- SC	Case II (b)- SC-BR	Case II (d)- SC-LS-BR	Case II (c)- SC-C-BR
60	0	0	0	0
120	0	0	0	0
180	0	0	0	0
240	0	0	0	0
300	0	0	0	0
360	0	1	2	1
420	1	5	5	5
480	2	6	6	6
540	3	7	9	9
600	4	8	11	13
660	5	9	16	18
720	8	11	19	22
780	9	13	23	113
840	10	15	26	114
900	12	17	30	117
960	13	20	34	119
1020	14	23	145	121
1080	15	27	147	125
1140	16	32	149	127
1200	17	36	151	131
1260	19	40	154	135
1320	21	44	156	138
1380	22	48	158	139
1440	23	51	159	142
1500	24	54	163	144
1560	25	56	166	146
1620	27	58	169	147
1680	28	60	173	148
1740	29	61	174	149
1800	30	62	175	150

Table 3 shows the settlement data for four different soil profiles when the flow inside the pipeline was non-continuous (5 s cyclic time interval).

**Table 3:** Settlement values for non-continuous water flow with 5 s cyclic time interval through four different soil profiles.

Time (s)	Settlement (mm)			
	Case III (a)- SC	Case III (b)- SC-BR	Case III (d)- SC-LS-BR	Case III (c)- SC-C-BR
60	0	0	0	0
120	0	0	0	0
180	0	0	0	0
240	0	0	0	0
300	0	0	0	0
360	0	1	3	1
420	1	5	6	5
480	2	7	8	7
540	4	8	10	10
600	5	9	13	15
660	6	10	18	20
720	9	12	20	119
780	10	14	24	120
840	11	16	28	122
900	13	19	34	123
960	14	21	149	125
1020	15	25	150	129
1080	16	28	152	133
1140	17	33	153	135
1200	18	38	154	137
1260	21	41	159	139
1320	22	45	161	142
1380	23	50	164	144
1440	24	52	166	149
1500	25	55	169	150
1560	27	57	175	151
1620	29	60	178	153
1680	30	62	179	155
1740	31	64	182	156
1800	32	66	183	156

Four cases for each condition shown in Tables 1, 2, and 3 (total 12 cases), as presented in [1] show the progression of settlement under various conditions with time.

## 2 Experimental Design, Materials and Methods

### 2.1 Data simulation setup

The software architecture for our data analysis was implemented using the R programming language. The R language has many benefits, such as compatibility with many operating systems and real-time implementation. The Origin Pro-tool was also used to cross-check the results of our analysis. In our study, the dataset has been re-organized and re-formated. The data is represented in different tables in order to illustrate the sinkhole mechanism under various subsurface soil profiles for different water flow conditions and time periods.

### 2.2 Experimental design and materials

The first stage of the experiment was to design the architecture in the laboratory. The overall architecture of the experimental setup is shown in [1]. Water was supplied to the system from a water tank (Tank 2) with a capacity of 227 L. The water passing through the pipeline was collected at the outlet, allowing measurement of the quantity of water seeping into the model box due to leakage. In total, 200 L of water was placed into the water tank for each case. As the pressure of the water flow at the inlet declines steadily with the water level inside the water tank, another water tank (Tank 1) was used to maintain the water level in Tank 2 to control the drop in water pressure, as shown by [1]. A solenoid valve was fixed at the bottom of each water tank to manually control the flow of water inside the pipeline. A PVC pipeline with an external diameter of 40 mm and an internal diameter of 36 mm was used. Artificial leakage was created by creating a hole in the pipeline, as shown by [1]. The model box used for the experiment had dimensions of 700 mm (width) × 600 mm (length) × 330 mm (height) with a hole at the center of the bottom for drainage. The different subsurface soil profiles considered in this study comprised combinations of bedrock, carbonate rock, cavities, sand, and clay.

### 2.3 Data analytic methods

To extract the dataset mentioned in Tables 1, 2, and 3, the following steps were followed:

1. Four soil profiles were considered: sandy clay (SC), sandy clay-bedrock (SC-BR), sandy clay-cavity-bedrock (SC—C-BR), and sandy clay-limestone-bedrock (SC-LS-BR).
2. In addition, two water flows in the pipeline were considered: continuous and cyclic, with two different cyclic time intervals.
3. The materials adopted for the 12 different soil profile models in the laboratory included sandy clay, limestone, sugar cubes, and gravel. Coarse aggregate (gravel) was used to represent bedrock, limestone powder was used to represent limestone, and sugar cubes were used to represent artificial cavities, as shown by [1].
4. Ali and Choi [1] represented the four different soil profiles under consideration in this study as A, B, C, and D. In each of the 12 cases, the soil was compacted in two layers, each 160 mm in thickness.
5. The soil was compacted manually with a brick of dimensions 20 cm × 6.3 cm × 5 cm, and 20 vertical blows were used for each case. To measure the settlement, a measuring tape was attached vertically to the center of each model box, and the reading was recorded in millimeters [1].
6. After the setup of the four different soil profiles, the water was continuously passed through in the first test. Settlement values were noted every 60 s for 1800 s for each of the four soil profiles.
7. Step 6 was repeated for a non-continuous flow of water with two different time intervals of 30 s and 5 s.
8. A camera was used to capture images of the experiment at the beginning and end of each case.

From the extracted dataset, the subsurface soil strata consisting of sandy clay, limestone, and bedrock (SC-LS-BR) are seen to dominate the sinkhole mechanism.

## Declaration of Competing Interest

The authors declare that they have no known competing financial interests or personal relationships which have or could be perceived to have influenced the work reported in this article.
